# Genome-Wide Identification, Classification and Expression Analysis of m^6^A Gene Family in *Solanum lycopersicum*

**DOI:** 10.3390/ijms23094522

**Published:** 2022-04-20

**Authors:** Hui Shen, Baobing Luo, Yunshu Wang, Jing Li, Zongli Hu, Qiaoli Xie, Ting Wu, Guoping Chen

**Affiliations:** Laboratory of Molecular Biology of Tomato, Bioengineering College, Chongqing University, Chongqing 400030, China; hbshenhui@163.com (H.S.); baobingluo@163.com (B.L.); wangyunshu@cqu.edu.cn (Y.W.); micy180605@163.com (J.L.); huzongli71@163.com (Z.H.); qiaolixie@cqu.edu.cn (Q.X.)

**Keywords:** tomato, m^6^A, m^6^Am, MT-A70, ALKBH, YTH

## Abstract

Advanced knowledge of messenger RNA (mRNA) *N*^6^-methyladenosine (m^6^A) and DNA *N*^6^-methyldeoxyadenosine (6 mA) redefine our understanding of these epigenetic modifications. Both m^6^A and 6mA carry important information for gene regulation, and the corresponding catalytic enzymes sometimes belong to the same gene family and need to be distinguished. However, a comprehensive analysis of the m^6^A gene family in tomato remains obscure. Here, 24 putative m^6^A genes and their family genes in tomato were identified and renamed according to BLASTP and phylogenetic analysis. Chromosomal location, synteny, phylogenetic, and structural analyses were performed, unravelling distinct evolutionary relationships between the MT-A70, ALKBH, and YTH protein families, respectively. Most of the 24 genes had extensive tissue expression, and 9 genes could be clustered in a similar expression trend. Besides, *SlYTH1* and *SlYTH3A* showed a different expression pattern in leaf and fruit development. Additionally, qPCR data revealed the expression variation under multiple abiotic stresses, and LC-MS/MS determination exhibited that the cold stress decreased the level of *N*^6^ 2′-O dimethyladenosine (m^6^Am). Notably, the orthologs of newly identified single-strand DNA (ssDNA) 6mA writer–eraser–reader also existed in the tomato genome. Our study provides comprehensive information on m^6^A components and their family proteins in tomato and will facilitate further functional analysis of the tomato *N*^6^-methyladenosine modification genes.

## 1. Introduction

*N*^6^-methyladenosine (m^6^A) is the most prevalent internal chemical decoration in eukaryotic mRNAs and non-coding RNAs [1,2]. As a dynamic and reversible post-transcriptional mark, m^6^A is installed by the writer complex containing METTL3, METTL14, and WTAP, and can be removed by erasers belonging to the ALKBH family [3,4,5,6]. Carrying m^6^A modification transcripts can be recognized by readers, such as YTH domain-containing proteins. m^6^A mediates its biological functions in affecting downstream RNA metabolism, including mRNA stability, splicing, translation efficiency, and nuclear export, by recruiting reader proteins [7,8,9]. Growing evidence suggests that m^6^A has essential biological functions. At the same time, false m^6^A modification affects cancer stem cell proliferation, embryo development, cell circadian rhythms, and cell fate decision [10,11,12,13,14]. Significant progress is being made in m^6^A detection technology, promoting m^6^A study deep into transcriptome level and single-base resolution [15,16,17,18]. Thus, m^6^A has been a very active and bourgeoning area of post-transcriptional epigenetic research in recent years. However, most studies of m^6^A are focused on human and other mammalian systems, and the relevant knowledge about the regulatory mechanisms of m^6^A in plants has been little.

In plants, most studies mainly of *Arabidopsis* have shown that m^6^A affects embryo development [19], stem cell fate determination [20,21], floral transition [22], trichomes, and leaf morphology [23,24,25]. More recent investigations in other species have demonstrated that m^6^A affects the fruit development and ripening of tomato [26,27] and strawberry [28], as well as the sporogenesis of rice [29]. Additionally, m^6^A also mediates plants’ biotic and abiotic stress responses [30,31,32,33]. Overall, accumulating evidence has dramatically enriched the knowledge of the biological functions of m^6^A in plant growth, development, and stress response, highlighting the biological importance of m^6^A modification. Preliminary identification of m^6^A modification components in different plant species by bioinformatics analysis is valid and ongoing [34,35,36]. However, systematic analysis of m^6^A methylation, demethylation, and recognition proteins in plants is extremely rare. Consequently, the directly relevant regulatory pathway of writer–eraser–reader remains largely unknown.

Adenine methylation modification as an essential epigenetic mark exists in both DNAs and RNAs of eukaryotes [37]. Eukaryotic N^6^A-MTases (N^6^A modification methylases) belong to three broad groups [38]. Group 1 contains the most widespread Ime4-like (also called MT-A70) clade, which subsequently radiates into six distinct eukaryotic sub-clades [38]. Three N^6^A-MTase clades (clades 1–3) are typified by METTL3, METTL14, and METTL4, respectively, and conserved in higher eukaryotes, whereas clades 4–6 exist in basal fungi, unicellular photosynthetic eukaryotes, and haptophyte algae [38]. METTL3 and METTL14 form a core heterodimer, catalyzing N^6^A methylation of specific positions in mRNAs, whereas METTL4 is likely to be a DNA methylase [39]. Compared with m^6^A methylase, FTO and ALKBH5 act as specific mRNA m^6^A demethylases, belonging to the ALKBH (ALKB homolog) subfamily of the Fe(II)/2-oxoglutarate (2OG) dioxygenase superfamily [40,41]. The human ALKBH family comprises nine members: ALKBH1-8 and FTO (FaT mass and obesity associated). The functional diversity may be due to their different substrate selectivity [42]. The ALKBH family in plants contains many members. Phylogenetic analysis showed that no orthologs of FTO are present, but that there are multiple copies of ALKBH5 orthologs in *Arabidopsis* [43]. Most of the known m^6^A readers are YTH domain-containing proteins. Compared to mammals, the YTH protein family is also expanded in plants. For example, there are 13 YTH domain-containing proteins in *Arabidopsis* and five in human [24]. Noticeably, in addition to m^6^A, single-strand DNA *N*^6^-methyladenine (6mA) modification has been found in mammals, and of which the known ssDNA 6mA catalytic enzymes also belong to the MT-A70, ALKBH, and YTH protein families, respectively [44,45,46,47]. However, ssDNA 6mA modification has not been reported in botany. The distinction between the m^6^A and 6mA modification enzymes is also neglected in evolutionary analysis. Thus, considering the expansion of family members and potential functional diversity, a more detailed evolutionary analysis is necessary to distinguish whether the putative m^6^A modification components act on RNAs or other substrates.

Tomato (*Solanum lycopersicum*) is an economically important fruit vegetable worldwide and a critical model plant for plant growth and fruit ripening. However, there is no comprehensive or systematic analysis of the m^6^A gene family in tomato. Additionally, the discoveries in mammals have rapidly enriched our knowledge of mRNA m^6^A and ssDNA 6mA. Thus, the renewed cognition applied to tomato may be a good entry point to investigate the m^6^A gene family and explore the evolutionary and functional differences. In the present study, we performed genome-wide identification, structural, evolutionary, expression pattern, and abiotic stress analyses of the m^6^A gene family in the tomato genome. A comprehensive and comparative analysis of the m^6^A gene family in tomato was first studied and discussed in this study. Our research aims to reveal the most covered area of *N*^6^-methyladenosine and its protein family in tomato, providing clues for studying its biological functions in the future.

## 2. Results

### 2.1. Genome-Wide Identification of m^6^A Gene Family in Tomato

To identify m^6^A components and their protein families in tomato, the amino acid sequences of m^6^A related proteins reported in *Arabidopsis thaliana* [43], including writers, erasers, and readers, were used as queries to perform BLASTP against the tomato genomic sequences both in NCBI and SGN servers. After removing the repeated sequences, a total of 27 putative candidates and their gene ID were obtained. Then the CD-Search and SMRAT programs were used to detect and confirm the presence of the conserved domain of each identified sequence. Three of the 27 candidates did not have the conserved and typical 2OG_Fe(II)_Oxy domain (CDD: pfam13532) (Figure S1) and were eventually removed. The remaining 24 candidate genes were renamed based on our subsequent evolutionary analysis. The amino acid sequence length, relative molecular weights (MWs), and isoelectric points (pIs) are listed in Table 1. In detail, the lengths of the listed proteins ranged from 253 (SlALKBH2) to 2196 (SlVIR) amino acids, and the corresponding range for MWs was 29.10–240.80 KDa. The predicted *pI* values ranged from 4.86 (SlALKBH7 and SlFIP37) to 9.02 (SlALKBH2). Among these genes, *SlFIP37*, *SlHAKAI*, and *SlVIR*, three putative catalytic subunits of the m^6^A methyltransferase complex, only had one copy in the tomato genome, respectively. However, the MT-A70, ALKBH, and YTH domain protein families consisted of multiple members.

### 2.2. Chromosomal Location and Collinearity Analysis of m^6^A Gene Family in Tomato

**Figure 1:**
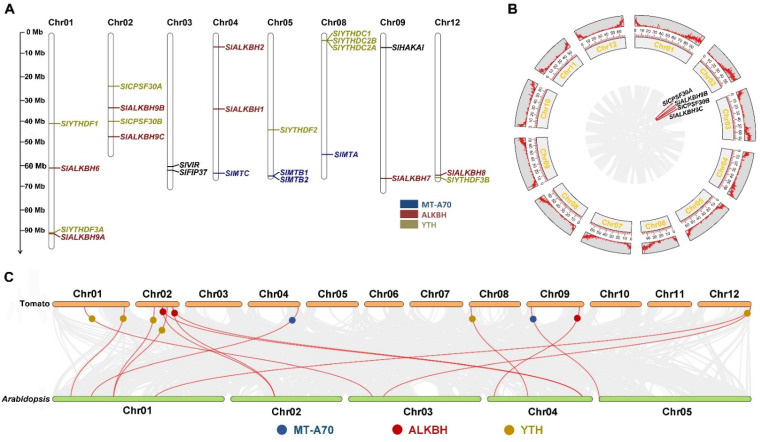
Chromosomal location and collinearity analysis of the m^6^A genes in tomato. (**A**) Locations of the m^6^A genes in tomato chromosomes. The scale at the left side of the figure is shown in Mb. The number of each chromosome is indicated at the top of the corresponding chromosome. (**B**) Synteny analysis of the m^6^A genes in tomato. The gray lines represent the collinearity result of the tomato genome, and the red lines represent the segmental duplication events. (**C**) Synteny analysis of the m^6^A genes between tomato and *Arabidopsis*. The gray lines represent the collinearity result between tomato and *Arabidopsis* genomes, and the red lines represent homologous gene pairs.

All 24 genes were distributed on eight chromosomes in tomato, and most of the genes were on the proximate or distal ends of the chromosomes. The MT-A70, ALKBH, and YTH family genes are highlighted in different colors (Figure 1A). Among these genes, *SlMTB1* and *SlMTB2* were adjacent on chr05, which may have been caused by a tandem duplication event. Similarly, another tandem duplication event was found on chr08, where *SlYTHDC1*/*SlYTHDC2A*/*SlYTHDC2B* were clustered into a subgroup (Figure 1A). The amino acid sequences between the proteins produced by these tandem duplications were highly conserved (Figure S2). Except for tandem duplication, segmental duplication was another driving force for gene family expansion. Genome-wide synteny analysis in tomato was analyzed, and two gene pairs, *SlCPSP30A*-*SlCPSF30B* and *SlALKBH9B*-*SlALKBH9C*, were identified as segmental duplication (Figure 1B). Therefore, both tandem duplication and segmental duplication appeared to involve in the expansion of the m^6^A gene family.

To further investigate the phylogenetic mechanisms of tomato m^6^A components and their protein families, a synteny analysis between tomato and *Arabidopsis* was constructed. Sixteen orthologous pairs consisting of 13 tomato genes and 12 *Arabidopsis* genes were identified (Figure 1C), which indicated the existence of these orthologous pairs prior to the divergence of *Arabidopsis* and tomato. Moreover, Ka/Ks calculation was performed to assess the extent and type of selective pressure of each gene pair. All five gene pairs were under purifying selection, and the earliest differentiation (*SlYTHDC1*/*SlYTHDC2A*) occurred 86.86 million years ago (Table 2).

### 2.3. Evolutionary and Structure Analyses of MT-A70 Family in Tomato

**Figure 2:**
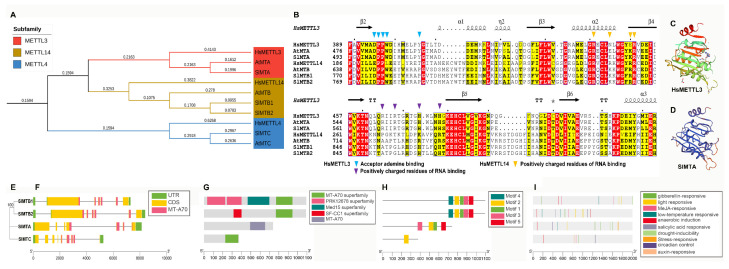
Evolutionary and structure analyses of the MT-A70 family in tomato. (**A**) Phylogenetic tree of MT-A70 family proteins from tomato, *Arabidopsis*, and human. (**B**) Sequence alignment of METTL3 and METTL14 subfamily proteins from tomato, *Arabidopsis*, and human. The secondary structural elements in the HsMETTL3 (PDB: 5l6d) MT-A70 domain are shown above. The colored triangles indicate key functional residues in human. (**C**,**D**) Three-dimensional structures of HsMETTL3 and SlMTA. (**E**) Phylogenetic tree of MT-A70 family proteins in tomato. (**F**) Gene structure analysis. The coding sequence (CDS), untranslated region (UTR), and MT-A70 domain are displayed in different colors, and the lines between boxes represent introns. (**G**,**H**) Conserved domain and motif analysis. (**I**) Locations of cis-elements in the 2 kb promoter sequences.

To analyze the evolutionary relationship among tomato MT-A70 family proteins, an unrooted phylogenetic tree was constructed using tomato, *Arabidopsis* MT-A70 sequences, and human reference sequences (Table S1). Phylogenetic analysis suggested that the MT-A70 proteins of tomato could be divided into three clades: METTL3 subfamily (SlMTA), METTL14 subfamily (SlMTB1 and SlMTB2), and METTL4 subfamily (SlMTC) (Figure 2A). Notably, compared to *Arabidopsis* and human, two copies of METTL14 orthologs were found in tomato, suggesting functional diversity or redundancy. A further multiple sequence alignment showed that many functional sites, including residues involved in AdoMet interactions and RNA binding, were conserved (Figure 2B), indicating that a core heterodimer catalyzing mechanism might be similar among tomato, human, and *Arabidopsis.* Considering that HsMETTL3 was identified as the core catalytic enzyme for m^6^A methylation, the three-dimensional structure of SlMTA, the ortholog of HsMETTL3, was constructed. The results showed that SlMTA and HsMETTL3 had a similar catalytic activity center (Figure 2C,D).

To further explore the structure and sequence characteristics of MT-A70 genes in tomato, a simpler neighbor-joining phylogenetic tree (Figure 2E) was constructed using full-length amino acid sequences to classify visualized analyses. *SlMTB1* and *SlMTB2* clustered in the same clade had a very similar gene structure, conserved domain, and conserved motifs, including their number and position (Figure 2F–H). These results indicate that *SlMTB1* and *SlMTB2* were evolutionarily conserved and different from *SlMTA* and *SlMTC*. Meanwhile, except for the MT-A70 protein domain (CDD: pfam05063), SlMTB1 had extra PRK12678 superfamily (CDD: PRK12678) and Med15 superfamily (CDD: pfam09606) domains, and SlMTB2 had an extra SF-CC1 superfamily (CDD: TIGR01622) domain (Figure 2G), indicating that SlMTB1 and SlMTB2 may participate in different regulatory pathways. In the conserved motif analysis, compared to SlMTB1 and SlMTB2, SlMTA lacked motif 4 and had the disarranged motif 3 in front of motif 2, whereas SlMTC only had motif 2 (Figure 2H). Finally, the 2kb potential promoter sequence upstream of the initiation codon was analyzed, and the cis-elements were visualized (Figure 2I), indicating that the MT-A70 family genes in tomato may respond to phytohormone, plant development-related, and abiotic stress. Detailed types, locations, and sequences of cis-elements are provided in Supplementary Materials Table S2.

### 2.4. Evolutionary and Structure Analyses of ALKBH Family in Tomato

**Figure 3:**
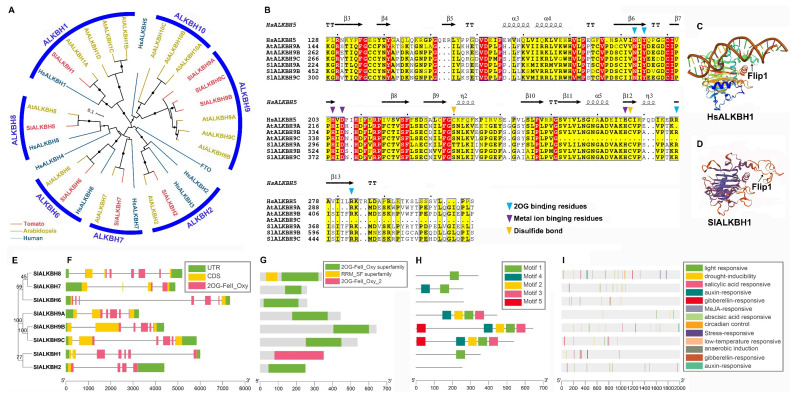
Evolutionary and structure analyses of the ALKBH family in tomato. (**A**) Phylogenetic tree of ALKBH family proteins from tomato, *Arabidopsis*, and human. (**B**) Sequence alignment of ALKBH9 subfamily proteins from tomato and *Arabidopsis*, and human HsALKBH5. The secondary structural elements in the HsALKBH5 (PDB: 4nj4) 2OG_Fe(II)_Oxy domain are shown above. The colored triangles indicate key functional residues in human. (**C**,**D**) Three-dimensional structures of HsALKBH1 (PDB: 6ie2) and SlALKBH1. (**E**) Phylogenetic tree of ALKBH family proteins in tomato. (**F**) Gene structure analysis. The coding sequence (CDS), untranslated region (UTR), and 2OG_Fe(II)_Oxy domain are displayed in different colors, and the lines between boxes represent introns. (**G**,**H**) Conserved domain and motif analysis. (**I**) Locations of cis-elements in the 2 kb promoter sequences.

To analyze the evolutionary relationship among ALKBH family proteins in tomato, an unrooted phylogenetic tree was constructed using tomato, *Arabidopsis* ALKBH sequences, and human reference sequences (Table S1). ALKBH proteins of tomato could be divided into seven subfamilies (Figure 3A). In tomato and *Arabidopsis*, ALKBH9 and ALKBH10 subfamily proteins were orthologs of the m^6^A methylase HsALKBH5. However, no homologue of the ALKBH10 subfamily protein was found in the tomato genome. Further multiple sequence alignment of HsALKBH5 and ALKBH9 subfamily proteins showed that many functional sites, including residues involved in 2OG and metal-binding, were conserved (Figure 3B), suggesting that SlALKBH9 subfamily proteins may have a similar catalyzing mechanism for methyl group removal as human HsALKBH5. Compared to m^6^A modification, HsALKBH1 was newly identified as the ssDNA (single-strand DNA) 6mA demethylase [45,46]. The three-dimensional structure of SlALKBH1, the ortholog of HsALKBH1, was constructed by homology modeling. Similarly, a functional “stretch-out” Flip1 structure also existed in SlALKBH1 (Figure 3C,D).

To further explore the structure and sequence characteristics of ALKBH genes in tomato, a simpler neighbor-joining phylogenetic tree (Figure 3E) was constructed using full-length amino acid sequences to classify visualized analyses. SlALKBH genes displayed relatively different gene structures, including the number and position of exons and introns (Figure 3F). Among the SlALKBH proteins, SlALKBH1 had a conserved domain of 2OG-Fe(II)_Oxy_2, whereas the others belonged to the 2OG-Fe(II)_Oxy superfamily (Figure 3G). In the conserved motif analysis, the SlALKBH9 subfamily had similar motifs 1–4, but SlALKBH9B and SlALKBH9C had an extra motif 5 (Figure 3H). Unexpectedly, no conserved motif was found in SlALKBH2 or SlALKBH6, and at the same time, SlALKBH1 and SlALKBH8 only showed conserved motif 1, and SlALKBH7 contained motif 1 and motif 4, suggesting a potential loss of function or functional differentiation of these proteins (Figure 3H). Finally, cis-elements of 2kb potential promoter sequence upstream of the start codon were analyzed and visualized (Figure 3I). Cis-elements were also classified into three categories: phytohormone responsive, plant development related, and abiotic stress responsive. The detailed information is provided in Supplementary Materials Table S2.

### 2.5. Evolutionary and Structure Analysis of YTH Family in Tomato

**Figure 4:**
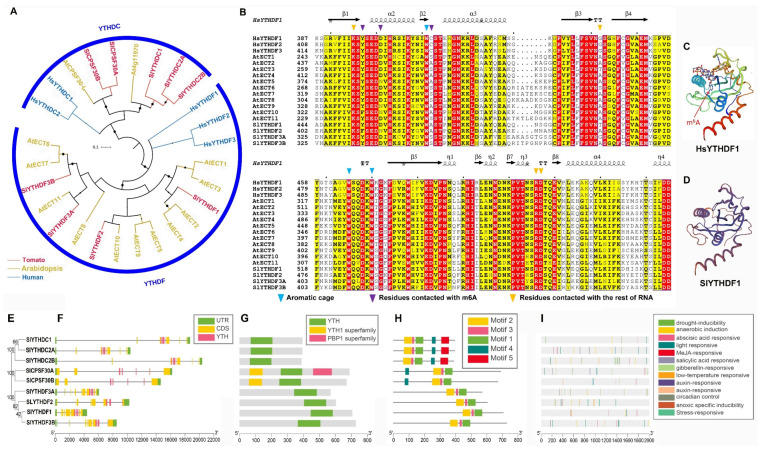
Evolutionary and structure analyses of the YTH family in tomato. (**A**) Phylogenetic tree of YTH family proteins from tomato, *Arabidopsis*, and human. (**B**) Sequence alignment of YTHDF subfamily proteins from tomato, *Arabidopsis*, and human. The secondary structural elements in the HsYTHDF1 (PDB: 4rcj) YTH domain are shown above. The colored triangles indicate key functional residues in human. (**C**,**D**) Three-dimensional structures of HsYTHDF1 and SlYTHDF1. (**E**) Phylogenetic tree of YTH family proteins in tomato. (**F**) Gene structure analysis. The coding sequence (CDS), untranslated region (UTR), and YTH domain are displayed in different colors, and the lines between boxes represent introns. (**G**,**H**) Conserved domain and motif analysis. (**I**) Locations of cis-elements in the 2kb promoter sequences.

To analyze the evolutionary relationship among YTH family proteins in tomato, an unrooted phylogenetic tree was constructed using the reference sequences of tomato, *Arabidopsis* YTH sequences, and human (Table S1). The YTH proteins of tomato could be divided into YTHDF and YTHDC subfamilies, and the YTHDC subfamily comprised two subclades: SlYTHDC and SlCPSF30 (Figure 4A). Compared to *Arabidopsis*, more YTH proteins belonged to the YTHDC subfamily in tomato (five in tomato and two in *Arabidopsis*), whereas fewer belonged to the YTHDF subfamily (four in tomato and 11 in *Arabidopsis*). Moreover, the AtECT1-4 subclade was functionally crucial in trichome and leaf morphology [23,24,25], whereas only one orthologous of tomato, SlYTHDF1, was classified in this subclade. In contrast, two orthologs of AtCPSF30-L existed in the tomato genome. Additional multiple sequence alignment of YTHDF subfamily proteins displayed that many functional sites, including residues involved in the aromatic cage, contact with m^6^A, and RNA binding, were conserved (Figure 4B), suggesting that SlYTHDFs might have a similar m^6^A read mechanism to human YTHDF proteins. Taking SlYTHDF1 as an example, through the three-dimensional structure, SlYTHDF1 and HsYTHDF1 shared a similar m^6^A binding structure (Figure 4C,D). Additionally, multiple sequence alignment of YTHDC subfamily proteins also displayed a conserved aromatic cage, suggesting the ability of the m^6^A read mechanism (Figure S3).

To further explore the structure and sequence characteristics of YTH genes in tomato, a simpler neighbor-joining phylogenetic tree (Figure 4E) was constructed using full-length amino acid sequences to classify visualized analyses. SlYTH genes clustered in the same clade shared a similar gene structure, including the number and position of exons and introns, and the distribution of the YTH domain on exons (Figure 4F). All SlYTHs had a typical YTH conserved domain (CDD: pfam04146) of similar length, whereas the positions of the YTH domain in different subclades were distributed on the C-terminal, middle site, and N-terminal, respectively (Figure 4G). Additionally, both SlCPSF30A and SlCPSF30B had the YTH1 superfamily domain (CDD: COG5084), and SlCPSF30A had the extra PBP1 superfamily domain (CDD: COG5180) (Figure 4G). All SlYTHs exhibited the conserved motifs 1–3 in the corresponding positions of the YTH domains. Moreover, the SlCPSF30 subclade proteins (SlCPSF30A and SlCPSF30B) had the extra conserved motif 4, whereas the SlYTHDC subclade proteins had the extra conserved motif 4 and motif 5 (Figure 4H). Together, these results indicate that the YTH family proteins were highly conserved in tomato, and that there was a slight evolutionary divergency in the subfamily or subclade. Finally, cis-elements were also analyzed and visualized (Figure 4I), and detailed information are listed in Supplementary Materials Table S2.

### 2.6. The Tissue Expression of m^6^A Genes and Their Family Genes in Tomato

**Figure 5:**
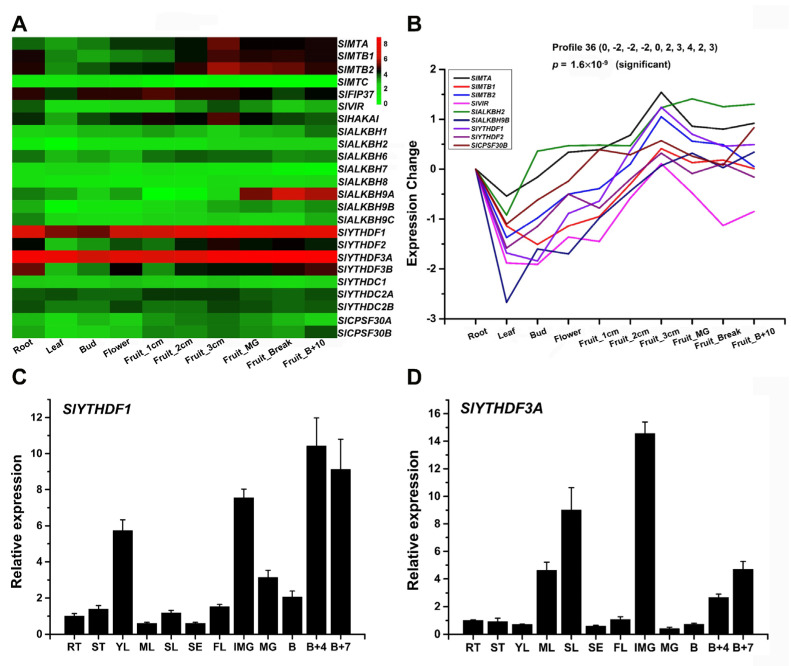
Heat map representation of the tomato m^6^A genes in various tissues. (**A**) Expression levels of tomato m^6^A genes in Heinz 1706 tomato based on transcriptome expression data. Each column represents a different tissue at different developmental stages of tomato. The bar on the right indicates normalized expression data from high to low (red to green). (**B**) STEM analysis. The expression data of each gene in root was normalized as 0. (**C**,**D**) Expression of *SlYTHDF1* and *SlYTHDF3A* in different tissues of Ailsa Craig tomato. RT, root; ST, stem; YL, young leaf; ML, mature leaf; SL, senescent leaf; SE, sepal; FL, flower; IMG, immature green; MG, mature green; B, breaker stage; B+4, 4 days after breaker stage; B+7, 7 days after breaker stage. Data are the mean ± SE of three independent experiments.

To investigate the expression patterns of m^6^A components in tomato and their family genes, the RNA-Seq data of 24 genes was downloaded from the previous tomato genome sequencing [48]. The expression data of 10 different tomato tissues (Root, Leaf, Bud, Flower, Fruit_1cm, Fruit_2cm, Fruit_3cm, Fruit_MG, Fruit_Break, and Fruit_B+10) at different developmental stages (Table S3) were used to construct a heatmap (Figure 5A). The expression profiles revealed that most of the tested genes displayed a broad expression range across all the organs and developmental stages, indicating that they were extensively involved in the growth and development of tomato. Compared with the other three genes in the MT-A70 family, *SlMTC* showed relatively lower expression levels, suggesting that *SlMTC* was nonfunctional or had temporal and spatial-specific expression pattern. Among the ALKBH family genes, *SlALKBH9A* exhibited tissue-specific expression and high expression levels in fruit-ripening stages. *SlYTHDF1* and *SlYTHDF3A* showed predominant expression among all 24 genes. Moreover, the same RNA-Seq data of the 24 genes (Table S3) was used for a mimical short time-series expression miner (STEM) analysis, and the results showed that nine of the 24 genes exhibited a significant trend of expression (Figure 5B), indicating that these genes might co-regulate the growth and development of tomato. Additionally, further RT-qPCR tests revealed that *SlYTHDF1* was highly expressed in newborn tissue (YL), and *SlYTHDF3A* was highly expressed in senescent tissues (ML and SL) at the vegetative growth stage (Figure 5C,D). *SlYTHDF1* and *SlYTHDF3A* showed a similar expression pattern at tomato fruit development stages, but *SlYTHDF1* had a higher mRNA abundance at the Fruit B+4 and B+7 stages.

### 2.7. Analysis of m^6^A Components and Their Family Genes under Abiotic Stress Treatments

**Figure 6:**
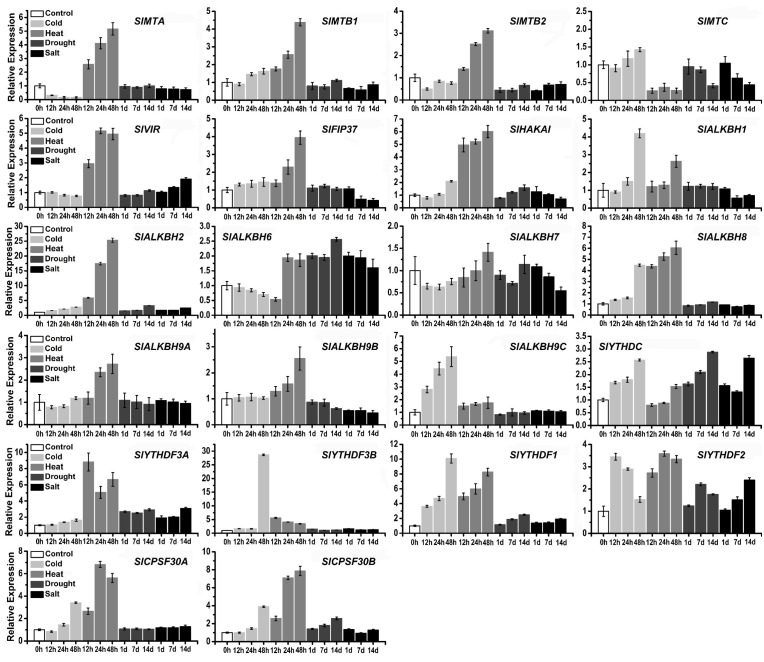
Expression profiles of tomato m^6^A genes in different abiotic stress by qPCR analysis. The 30-day-old tomato seedlings were treated under cold and heat conditions for 12, 24, and 48 h. Tomato seedlings were treated under drought and salt condition for 1, 7, and 14 d. Tomato seedlings without treatment was considered the control. Each value represents the mean ± SE of three replicates.

Recent evidence demonstrates that m^6^A modification is involved in plant responses to various abiotic stresses. In this study, four abiotic stress treatments, including heat, cold, salt, and drought, were used to detect the response of m^6^A modified genes. m6A writers (*SlFIP37*, *SlVIR*, and *SlHAKAI*) and MT-A70 family genes were significantly upregulated by heat stress except for *SlMTC*, whereas the other three kinds of stress treatments did not cause significant changes in gene expression levels (Figure 6). However, the expression changes of ALKBH members were more diverse under different treatments. Compared with other abiotic stress treatments, *SlALKBH2* showed a significant sensitive response to heat treatment. Heat stress treatment significantly upregulated the expression of *SlALKBH2*, *SlALKBH6*, *SlALKBH8*, and *SlALKBH9A*. The expression of *SlALKBH9C* was immediately increased by cold stress treatment, whereas *SlALKBH1* and *SlALKBH8* were significantly upregulated after cold treatment for 48 h. Salt and drought stress enhanced the expression of *SlALKBH6* and *SlALKBH9A*, but downregulated *SlALKBH9B*. The YTH family genes were essential m^6^A mark decoders, and most of them were upregulated in response to heat stress, except for *SlYTHDC*. In this study, *SlYTHDC1*, *SlYTHDC2A*, and *SlYTHDC2B* were represented by *SlYTHDC* and detected together through the co-source region. The results showed that *SlYTHDC* had a slight upregulation under cold and salt treatment. Cold stress also upregulated the expression of several readers, including *SlYTHDF1*, *SlYTHDF3A*, *SlYTHDF3B*, *SlCPSF30A*, and *SlCPSF30B*, whereas salt and drought stress induced only slight changes. The expression variation under different treatments indicated that m^6^A components and their family genes were involved in complex abiotic stress responses in tomato.

### 2.8. Detection of RNA Modifications by LC-MS/MS

**Figure 7:**
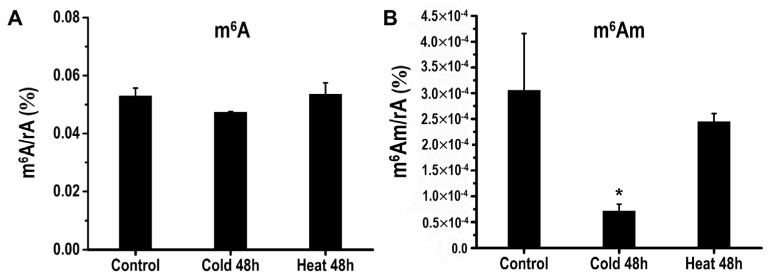
The amount of m^6^A and m^6^Am in tomato leaves by LC-MS/MS analysis. The changes in (**A**) m^6^A and (**B**) m^6^Am contents under cold and heat treatment. The 30-day-old tomato seedlings were treated under cold and heat conditions for 48 h. * Refer to significant differences with *p* < 0.05 compared to the control.

LC-MS/MS is an effective method for detecting modified nucleotides [49]. Considering that most genes tested by RT-qPCR are inclined to respond to cold and heat stresses, heat and cold treatment materials were used to perform the LC-MS/MS test and untreated material was the control. In total, 55 kinds of RNA modifications were tested, of which 30 had readable values, including m^6^A, m^6^Am, m^1^A, m^5^C, and ac4C (Table S4). Intriguingly, the control leaf material had an m^6^A/rA rate of 0.053% in total RNA, whereas heat treatment did not affect the overall modification level of m^6^A/rA (0.053% on average), and cold treatment only slightly reduced the m^6^A/rA ratio (0.047% on average) (Figure 7A). The m^6^A/rA ratio showed no significant changes under cold and heat stress treatment, which might have been due to partial methylation of the transcripts or RNA molecules and demethylation of the others. More unexpectedly, the m^6^Am (*N*^6^, 2′-O dimethyladenosine), a cap-specific terminal *N*^6^-methylation of RNA that can regulate RNA stability or the translation efficiency, was significantly reduced under cold stress treatment (Figure 7B). Usually, when adenosine is transcribed as the first cap-adjacent nucleotide, adenosine can be methylated both at the 2′ -hydroxyl and N6 positions, thus generating m^6^Am [50,51,52,53]. Unlike m^6^A, which is an internal modification, m^6^Am is a terminal modification at the transcription start site of capped mRNAs, hinting at a cap-specific post-transcriptional regulatory mechanism. Noticeably, the phosphorylated CTD interacting factor 1 (PCIF1) is newly identified as an m^6^Am methyltransferase in mammals [50,51,52,53], yet the catalytic component and functions of m^6^Am in plants are still unknown.

## 3. Discussion

Previously, 26 putative m^6^A proteins were obtained from the Tomato Genome Database and only used to construct the phylogenetic tree to analyze the evolutionary relationship among the plant kingdoms [34]. The 26 putative proteins, including MT-A70, ALKBH, and YTH family proteins, were named only by the relative chromosomal locations. In this study, a total of 24 putative m^6^A genes in tomato, including potential m^6^A writers, erasers, readers, and their family genes, were identified by BLASTP analysis. Two putative ALKBH family genes identified in the previous report were removed because of the absence of the 2OG-Fe(II)-Oxy conserved domain in their full-length protein sequences with both CDD-search and SMART analysis. Thus, a total of eight ALKBH family genes were identified in this study, a similar result as another study [27]. Moreover, we renamed 24 genes according to our phylogenetic analysis, which will facilitate further functional analysis of these genes. Among 24 genes, the MT-A70, ALKBH, and YTH families are each composed of multiple genes. Thus, we further analyzed the evolutionary relationships and potential functional divergences within these protein families.

Gene duplications are considered one of the main driving forces of genetic evolution [54]. Segmental, tandem replications and transposition events represent three main evolutionary patterns [55]. Land plant genomes encode a single copy of MTA and MTC, whereas multiple copies of MTB occur in several species [43]. The same evolutionary pattern was found in tomato. Two copies of MTB (*SlMTB1* and *SlMTB2*) were adjacently distributed on chr05, which may have been due to the tandem replication (Figure 1A). Another tandem replication event occurred in the YTHDC subfamily proteins, including SlYTHDC1, SlYTHDC2A, and SlYTHDC2B (Figure 1A). Compared to tandem duplication, our synteny analysis also showed gene duplication in the segmental manner (Figure 1B). These results reveal the dynamic expansion of the m^6^A gene family and potential functional diversity or redundancy in tomato.

As reported, the m^6^A methyltransferase complex seems to be conserved between mammals and plants, except when the plant m^6^A “writer” complex includes the orthologs of ZC3H13, RBM15, and RBM15B, which awaits further investigation [56]. The components of the m^6^A writer complex were similar between tomato and *Arabidopsis*, including the orthologs of MTA, MTB, FIP37, VIR, and HAKAI (Table 1). However, two orthologs of MTB were found in the tomato genome, and different conserved domains were predicted between SlMTB1 and SlMTB2 (Figure 2G). These results hint at a more complex “writer” mechanism of m^6^A in tomato. In the ALKBH family, SlALKBH9A (called SlALKBH2 in Zhou [27]) was identified as the m^6^A demethylase and affected fruit ripening by regulating the DNA demethylase SlDML2 [27]. However, it remains unknown how m^6^A demethylase affects tomato growth and development, and whether it regulates fruit ripening through other pathways. Intriguingly, our evolutionary and structure analyses revealed a visible evolutionary divergence among ALKBH family genes in tomato (Figure 4). Among the ALKBH family, SlALKBH9B and SlALKBH9C, a segment duplication pair, were classed into the same subclade with SlALKBH9A, suggesting a potential m^6^A demethylation activity. Previously, ALKBH proteins, except for ALKBH5 in human, displayed functional diversity [42]. For example, HsALKBH1 can remove methyl groups from DNA and RNA, HsALKBH2 has DNA repair activity, HsALKBH7 is involved in fatty acid metabolism and programmed necrosis, and HsALKBH8 is required for 5-methoxycarbonylmethyluridine (mcm5u) biogenesis in tRNA. Thus, our evolutionary analysis will facilitate the identification of new m^6^A demethylases in tomato. On the other hand, our results lay a foundation for exploring the function differentiation of ALKBH family members in tomato. Compared to mammals, the number of YTH domain proteins in tomato was greatly expanded (Figure 4A), indicating a more complex regulatory mechanism or functional redundancy, which has been well discussed in a previous study [36].

Both mRNA (*N*^6^-methyladenosine (m^6^A)) and DNA (*N*^6^-methyladenine (6mA)) has been detected in eukaryotes [37]. In plants, two studies revealed that 6mA widely occurs in the *Arabidopsis* and rice genomes, and 6mA as a DNA marker was associated with regulating gene expression [57,58]. However, no studies on 6mA in tomato have been reported so far. In mammals, the 2-oxoglutarate-dependent oxygenase ALKBH1 acts as a nuclear eraser of *N*^6^-mA in single-stranded and transiently unpaired DNA [45,46]. In general, the transient local unwinding of dsDNA occurs during transcription, replication, recombination, and DNA repair. Notably, in our study, unlike *Arabidopsis*, only one copy of the ALKBH1 ortholog was present in the tomato genome, whereas *Arabidopsis* comprised four copies of ALKBH1 orthologs (Figure 2). Additionally, the three-dimensional (3-D) model of SlALKBH1 exhibited a similar spatial structure as mammalian HsALKBH1, especially in harboring a functional “stretch-out” Flip1 structure (Figure 2). The “stretch-out” Flip1 of ALKBH1 is a unique functional structure that generated the catalytic activity of 6mA demethylase on ssDNA [45]. These results suggest that SlALKBH1 might have a similar demethylation activity of 6mA on ssDNA. More recent investigations revealed that the METTLL3-14 MTase complex and YTHDC1 could bind to 6mA on ssDNA in mammals [44,47]. Altogether, a regulating model of writer–reader–eraser targeting 6mA on ssDNA has been identified in mammals. However, whether the 6mA in ssDNA or the potential 6mA modification enzyme exists in tomato remains unknown. Our results show that SlALKBH1 and HsALKBH1 were clustered in the ALKBH1 subfamily, and they shared a similar 3D structure, suggesting SlALKBH1 as a potential 6mA demethylase in ssDNA.

Most m^6^A components and their family genes showed a broad expression pattern, suggesting that they play a broad and essential regulatory role in the growth and development of tomato. m^6^A methylases had similar expression patterns (Figure 5A), which is consistent with the mechanism that multiple methylases form a writer complex to catalyze m^6^A. However, *SlVIR* showed a relatively low expression compared with other methylases, suggesting that *SlVIR* might mediate a more specific regulatory pathway. Interestingly, a recent study of the *vir* mutant showed that the level of m^6^A was obviously reduced, especially in the 3ʹ untranslated region (3ʹ-UTR) [59]. Among the ALKBH family in tomato, *SlALKBH9A* was tissue-specific and expressed during fruit ripening (Figure 5A), which turned out to be related to fruit ripening [27]. In the YTH family, *SlYTH1* and *SlYTH3A* showed predominant expression among the 24 genes (Figure 5A). Moreover, our additional RT-qPCR tests revealed that *SlYTH1* was highly expressed in newborn tissue (YL), and *SlYTH3A* was highly expressed in senescent tissues (ML and SL) (Figure 5CD). In previous reports, *AtECT2* was highly expressed in rapidly growing tissues [25], and the delayed first true leaf emergence in *ect2 ect3* double mutants and the mutation of *AtECT4* enhanced this phenotype [23]. Phylogenetic analysis showed that SlYTH1 and AtECT2/3/4 belong to the same subclade of the YTHDF subfamily, whereas SlYTH3A belongs to another subclade of the YTHDF subfamily (Figure 4A). These results hint that SlYTH1 and SlYTH3A are functionally different and co-regulate the entire development process of leaves. Additionally, nine genes were clustered in a similar expression trend (Figure 5B), suggesting the potential synergistic regulation of writers, erasers, and readers in tomato growth and development.

In plants, m^6^A modification is also thought to be involved in response to abiotic stresses. However, whether the m^6^A gene responds to abiotic stress in tomato remains unknown. In the present study, cis-elements on 2kb potential promoter sequences of 24 genes were analyzed, suggesting that these genes might respond to phytohormones, plant development-related signals, and abiotic stress (Table S3). Moreover, we found that the expression levels of m^6^A genes were generally more responsive to cold and heat treatments in tomato (Figure 6). Intriguingly, tomato leaf and *Arabidopsis* had similar m^6^A content (0.053% in tomato; 0.05–0.07% in *Arabidopsis*), whereas cold and heat treatment did not affect the modification level of m^6^A in total RNA (Figure 7A). This unexpected phenomenon may have been due to the increased levels of m^6^A modification in some parts of the transcripts and RNAs and decreased levels in others. For example, the m^6^A level of 1805 transcripts was decreased, and 978 transcripts were increased in tomato anthers induced by low-temperature stress [60]. More recently, the new field of study investigating mRNA modification is m^6^Am (*N*^6^, 2′-O dimethyladenosine), a cap-specific terminal *N*^6^-methylation of RNA and regulating RNA stability or the efficiency of translation. Interestingly, compared to the control, the level of m^6^Am was significantly reduced under cold stress (Figure 7B), suggesting that m^6^Am responded to low-temperature stress in tomato leaves. As reported, PCIF1 KO cells with dramatically decreased levels of m^6^Am showed strong sensitivity to H_2_O_2_ treatment [51], indicating that m^6^Am plays a regulatory role in response to oxidative stress. Together, although the relevant knowledge about the regulatory mechanisms of m^6^Am remains largely unknown, our results can provide evidence for dynamic modification of m^6^Am in botany, highlighting the biological role of m^6^Am in responding to abiotic stress.

## 4. Materials and Methods

### 4.1. Identification of m^6^A Components and Their Protein Families in Tomato

To identify all the m^6^A components and their family proteins in the tomato genome, the amino acid sequences of m^6^A-related proteins reported in *Arabidopsis thaliana* [43], including writers, erasers, and readers, were used as queries to perform BLASTP against the tomato genomic sequences both in the National Center for Biotechnology Information (NCBI, https://www.ncbi.nlm.nih.gov/ (accessed on 19 August 2021)) and Sol Genomics Network (SGN, https://solgenomics.net/ (accessed on 19 August 2021)) websites. After removing the repeated sequences, a total of 27 putative candidates, the gene IDs, and the full-length amino acid sequences were obtained. Then, the CD-Search (https://www.ncbi.nlm.nih.gov/Structure/cdd/cdd.shtml (accessed on 19 August 2021)) and SMRAT (http://smart.embl-heidelberg.de/ (accessed on 19 August 2021)) programs were used to detect and confirm the presence of conserved domains in each identified sequence. The molecular weight (MW) and isoelectric points (*pI*) were predicted via the ExPaSy (http://web.expasy.org/protparam/ (accessed on 19 August 2021)) tool.

### 4.2. Chromosome Location, Synteny Analysis, and Ka/Ks Calculation

The reference genomes of tomato and *Arabidopsis thaliana* used in this article were assembly SL3.0 and TAIR10.1. The tomato and *Arabidopsis thaliana* genome sequences and annotation files (GFF, FASTA suffix files) were downloaded from the NCBI database (https://www.ncbi.nlm.nih.gov/genome/ (accessed on 20 August 2021)). The length of each chromosome and the positional information of the m^6^A components and their family genes on the chromosomes were extracted from the tomato GFF file by TBtools [61]. MapChart software [62] was used to draw the schematic diagram of chromosomal length scale and chromosomal locations. For the synteny analysis, the gene duplication landscape was obtained using MCScanX [63], and a syntenic map was generated and visualized by TBtools. The putative duplicated genes were highlighted by connection lines. The value of Ka and Ks were calculated by “simple Ka/Ks_calculation” in TBtools, and the formula T = Ks/r was used to calculate the divergence time [64].

### 4.3. Alignment and Phylogenetic Analysis

Multiple alignment of selected full-length amino acid sequences was aligned with default parameters using MAFFT v7 [65]. The secondary structure was annotated in the alignment using the ENDscript server [66]. Alignment results were used to construct a neighbor-joining (NJ) tree using MEGA11 [67] with Poisson correction, partial delete, and 1000 bootstrap replicates. The bootstrap values (>50%) on the major branches were shown. Interactive Tree Of Life (iTOL) v6.3 (https://itol.embl.de/ (accessed on 20 August 2021)) was used to visualize the phylogenetic tree. The secondary structures of HsMETTL3 (PDB ID: 5L6D), HsALKBH5 (PDB ID: 4NJ4), and HsYTHDF1 (PDB ID: 4RCJ) were downloaded from the NCBI structure database (https://www.ncbi.nlm.nih.gov/structure (accessed on 20 August 2021)). The protein sequences and their identifier (ID) used in this article are supported in Supplementary Materials Table S1.

### 4.4. Structure Construction by Homology Modeling

The selected full-length amino acid sequence was queried against the SWISS-MODEL server (https://swissmodel.expasy.org/ (accessed on 22 August 2021)) to search for templates, and the best template with a similar amino acid sequence and known 3D (three-dimensional) structure was used to Build Model. The 3D structure templates used in this article were HsMETTL3 (PDB ID: 5L6D), HsALKBH1 (PDB ID: 6IE2), and HsYTHDF1 (4RCJ). All the 3D structures of the template and homology modeling results were downloaded with cartoon type form SWISS-MODEF.

### 4.5. Gene Structure, Conserved Domain, Conserved Motif, and Cis-element Analyses

The information on gene lengths and structure was extracted from the tomato GFF file (assembly SL3.0) and was subsequently visualized by TBtools. The conserved domains of multiple full-length protein sequences were analyzed in the Batch CD-Search program (https://www.ncbi.nlm.nih.gov/cdd/ (accessed on 23 August 2021)), and then the output file (txt suffix) was downloaded. The conserved motifs were analyzed using MEME Suite software [68], and the output file (xml suffix) was downloaded. The 2000 bp promoter sequence upstream of start codon (ATG) was extract from the FASTA file of the tomato genome by TBtools. Then, the sequences were submitted to PlantCARE (http://bioinformatics.psb.ugent.be/webtools/plantcare/html/ (accessed on 23 August 2021)) to identify cis-elements (CREs), and the output file (tab suffix) was downloaded. The data set of cis-elements was simplified manually. Finally, all the downloaded files together with the tomato GFF file and the phylogenetic tree (nwk suffix) were submitted to TBtools for visualized analyses.

### 4.6. Digital Gene Expression and STEM Analysis

To obtain the expression profile of m^6^A components and their family genes in tomato, the RNA-Seq data based on the locus/gene names of SGN were analyzed. We downloaded the RNA-Seq data from various tissues (Tomato Genome, 2012), including root, leaf, bud, flower, and fruit (six developmental stages). RNA-Seq data were normalized using log2 (reads per kilobase of per million mapped reads (RPKM)) values. Visualization of expression profiling was performed by using the OmicStudio tools (https://www.omicstudio.cn/tool/ (accessed on 23 August 2021)). The RNA-Seq data of 24 genes were also used to perform a mimical STEM analysis [69]. To obtain the expression trend of each gene in 10 tissues, the expression data of the root were normalized as 0 to analyze the expression levels of other tissues relative to roots. Correlation analysis of expression trend was detected by *p*-value.

### 4.7. Plant Materials, Growth Conditions, and Stress Treatments

The tomato (*Solanum lycopersicum*) cultivar Ailsa Craig was obtained from the Laboratory of the Molecular Biology of Tomato, Bioengineering College, Chongqing University, Chongqing, China. Seedlings were grown in a controlled greenhouse with a 16 h day (25 °C)/8 h night (18 °C) cycle, 250 µmol photons m^−2^ s^−1^ light intensity, and 70% relative humidity, and managed routinely. Almost four-week-old seedlings were used for abiotic stress treatments. These stress conditions were set to evaluate the gene expression pattern, including cold stress (4 °C), heat stress (37 °C), salt stress (300 mmol/L NaCl), and drought stress (20% PEG 6000). The plants were separately treated by salt and drought stresses for 14 days. At 0, 1, 7, and 14 days, leaf samples under each treatment were obtained with three independent biological replicates. The plants were separately treated by cold and heat stresses for 48 h. At 0, 12, 24, and 48 h, leaf samples under each treatment were obtained with three independent biological replicates. After that, all samples that we used were immediately frozen in liquid nitrogen and kept at −80 °C for RNA extraction.

### 4.8. Total RNA Extraction and qPCR Analysis

Total RNAs were extracted using Trizol reagent (Invitrogen, Carlsbad, CA, USA) according to the manufacturer’s instructions. Genomic DNA contamination was erased by DNase digestion (Promega, Madison, WI, USA). The first-strand cDNA synthesis was performed using 1 μg of total RNAs by M-MLV reverse transcriptase (Promega, Madison, WI, USA). The RT-qPCR analysis was performed on a CFX96 Touch™ Real-Time PCR Detection System (Bio-Rad, Hercules, CA, USA). The PCR amplification parameters were as follows: 95 °C for 2 min, followed by 40 cycles (95 °C for 15 s and 60 °C for 40 s) and one cycle (95 °C for 15 s and 60 °C for 15 s). The *SlCAC* gene of tomato was used as an internal standard [70], and the 2^−ΔΔCt^ method was used to perform the relative gene expression level analysis [71]. All the experiments were performed in three biological triplicates with three technical replicates. All the primers used were designed by Primer 5.0 software and are shown in Table S5.

### 4.9. Detection of RNA Modifications

RNA modification contents were detected by MetWare (http://www.metware.cn/ (accessed on 10 October 2021)) based on the AB Sciex QTRAP 6500 LC-MS/MS platform. Significantly regulated metabolites between groups were determined by t-test *p*-value and absolute Log2FC (fold change).

### 4.10. Data Analysis

The mean values of the data are presented as mean ± SE (standard error). The Origin 8.0 software (available online: https://www.originlab.com/ (accessed on 3 December 2021)) was used to perform the data analysis, and mean differences were determined to be significant by *t*-test (* *p* < 0.05).

## 5. Conclusions

In the present study, a comprehensive and systematic analysis of the m^6^A gene family in tomato, including writers, erasers, and readers, was first conducted. A total of 24 genes were identified and renamed to better understand the underlying gene functions. The chromosomal distribution and synteny relationships, phylogenetic relationships, secondary and 3D structures, expression patterns, and responses to abiotic stresses of the putative m^6^A genes were characterized. Gene duplications were found in the MT-A70, ALKBH, and YTH protein families of tomato, which might directly cause the expansion of protein families and result in potential functional diversity or redundancy. Comparative phylogenetic tree analyses among tomato, *Arabidopsis*, and human were constructed and classed into subclades, which was helpful to distinguish the function of different subfamilies. Our results show that the orthologs of mammalian ssDNA 6mA proteins existed in the tomato genome, and SlALKBH1 exhibited a similar functional structure to HsALKBH1. These results provide evidence of the potential ssDNA 6mA modification in plants. The expression patterns showed that most of the genes had extensive tissue expression, and a mimical STEM was performed to analyze the similar expression cluster. *SlYTH1* and *SlYTH3A* showed predominant expression, and qPCR test results revealed different tissue expression. Additionally, qPCR data revealed that the m^6^A family genes responded to multiple abiotic stresses. Instead of m^6^A, the content of m^6^Am, a cap-specific terminal *N*^6^-methylation of RNA, was significantly decreased in the total RNA of tomato leaf under cold treatment. These results also provide evidence of the potential m^6^Am modification in plants. In general, our study provides comparative information among m^6^A, 6mA, and m^6^Am, which enables a better understanding of the *N*^6^-methyladenosine and lays the foundation for research into the comprehensive functional characteristics in the *N*^6^-methyladenosine modification in tomato. Furthermore, our bioinformatics and evolutionary analysis will be helpful for better understanding the underlying evolutionary relationship of the *N*^6^-methyladenosine modification in higher plants.

## Figures and Tables

**Table 1:** Characteristics of the m^6^A genes identified in tomato.

Protein Family	Gene Name	Gene ID	Protein Length (aa)	Molecular Weight (KD)	Theoretical *pI*
MT-A70	*SlMTA*	Solyc08g066730.3	739	81.47	6.43
	*SlMTB1*	Solyc05g056210.2	1094	122.62	6.34
	*SlMTB2*	Solyc05g056220.2	1091	121.98	6.39
	*SlMTC*	Solyc04g079950.3	376	48.09	6.73
ALKBH	*SlALKBH1*	Solyc04g045590.3	354	39.91	5.59
	*SlALKBH2*	Solyc04g015080.3	253	29.10	9.02
	*SlALKBH6*	Solyc01g057570.3	261	29.47	6.70
	*SlALKBH7*	Solyc09g074920.3	259	29.47	4.86
	*SlALKBH8*	Solyc12g096230.2	342	38.59	6.32
	*SlALKBH9A*	Solyc01g104130.3	445	50.47	8.76
	*SlALKBH9B*	Solyc02g062180.3	643	71.18	5.87
	*SlALKBH9C*	Solyc02g083960.3	538	60.30	6.37
YTH	*SlYTHDF1*	Solyc01g028860.3	706	77.22	6.95
	*SlYTHDF2*	Solyc05g032850.3	604	65.97	5.31
	*SlYTHDF3A*	Solyc01g103540.3	570	63.29	8.49
	*SlYTHDF3B*	Solyc12g099090.2	728	79.40	6.05
	*SlYTHDC1*	Solyc08g007740.2	395	44.21	6.39
	*SlYTHDC2A*	Solyc08g007760.3	394	44.27	6.10
	*SlYTHDC2B*	Solyc08g007750.3	389	43.16	6.17
	*SlCPSF30A*	Solyc02g021760.3	689	75.93	6.24
	*SlCOSF30B*	Solyc02g070240.3	671	73.77	6.10
	*SlFIP37* *	Solyc03g112520.3	342	38.64	4.86
	*SlVIR* *	Solyc03g020020.3	2196	240.80	5.49
	*SlHAKAI* *	Solyc09g013120.3	424	46.47	6.80

* Represent the putative catalytic subunits of the m^6^A methyltransferase complex.

**Table 2:** Estimated Ka/Ks ratios of the duplicated m^6^A genes and their divergence time in tomato.

Duplicated Gene Pair	Ka	Ks	Ka/Ks	Duplication Type	Selection	Time (MYA)
*SlMTB1/SlMTB2*	0.083561	0.291480	0.286679	Tandem	Purifying	9.71
*SlYTHDC1/SlYTHDC2A*	0.793902	2.605968	0.304647	Tandem	Purifying	86.86
*SlYTHDC2A/SlYTHDC2B*	0.879281	2.117476	0.415249	Tandem	Purifying	70.58
*SlALKBH9B/SlALKBH9C*	0.187651	0.724894	0.258866	Segment	Purifying	24.16
*SlCPSF30A/SlCPSF30B*	0.11570	0.585231	0.197709	Segment	Purifying	19.50

Ks: the number of synonymous substitutions per synonymous site; Ka: the number of non-synonymous substitutions per nonsynonymous site; MYA: million years ago.

## Data Availability

Data are contained within the article or Supplementary Material.

## Supplementary Materials

The following supporting information can be downloaded at: https://www.mdpi.com/article/10.3390/ijms23094522/s1.
