# Supplementary material for: Genome-Wide Identification, Classification and Expression Analysis of m6A Gene Family in Solanum lycopersicum
Source: Int J Mol Sci. 2022 Apr 20;23(9):4522. doi: 10.3390/ijms23094522 (PMC9100520; doi:10.3390/ijms23094522)
Supplement: Supplementary file 1 [file ijms-23-04522-s001.zip › Supplementary Figures.pdf]

Genome-Wide Identification, Classification and Expression Analysis of m⁶A Gene Family in *Solanum lycopersicum*

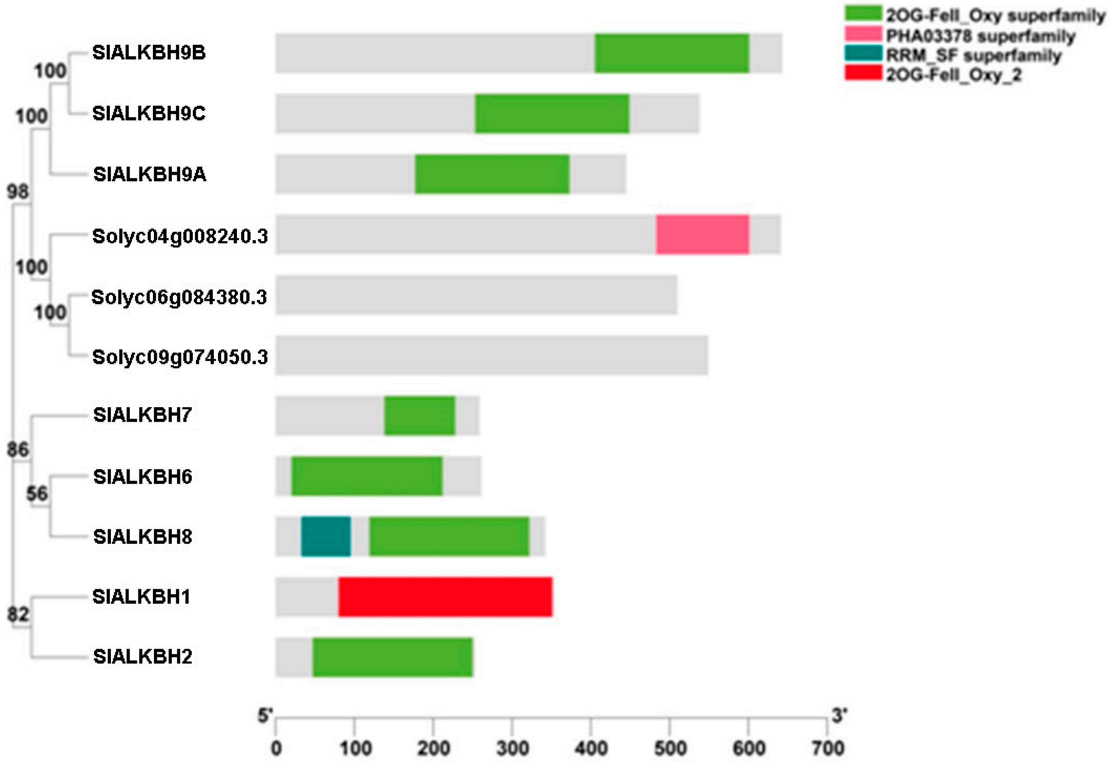

Figure S1. Conserved domains of putative ALKBH family proteins in tomato. Total 11 putative SlALKBH proteins were identified by BLASTP. Neighbor-joining (NJ) tree was constructed using MEGA11 with poisson correction, partial delete, and 1000 bootstrap replicates. Conserved domains in full length amino acid sequences were searched using the Batch CD-Search program and visualized by TBtools.

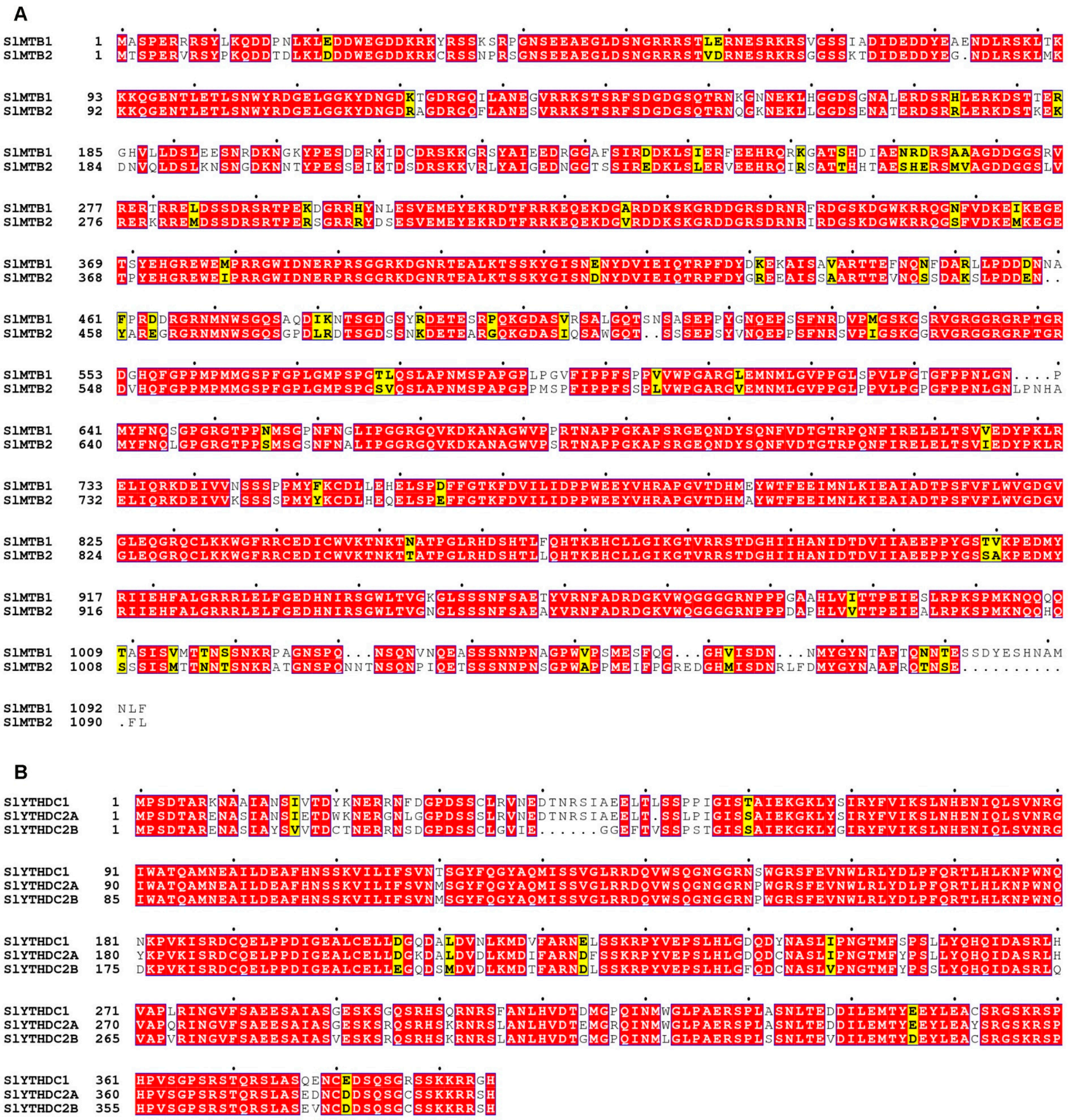

Figure S2. Sequence alignments of tandem duplication pairs. (A) Sequence alignments of SlMTB1 and SlMTB2. (B) Sequence alignments of SlYTHDC1, SlYTHDC2A and SlYTHDC2B. Full-length amino acid sequences were aligned by MAFFT v7, and the alignment was visualized in the ENDscript serve.

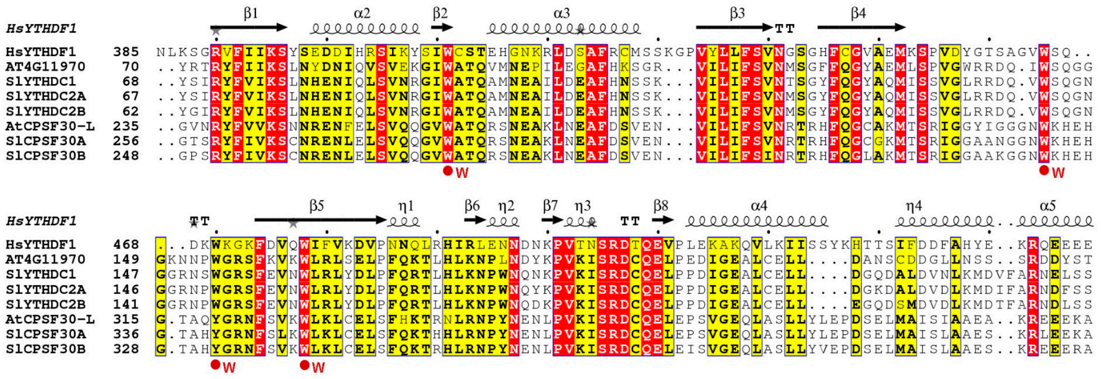

Figure S3. Multiple alignment of SlYTHDC subfamily proteins. Multiple alignment was aligned with default parameters using MAFFT v7. The secondary structure of HsYTHDF1 (PDB: 4rcj) was annotated in the alignment using the ENDscript server.
